# Sporadic Creutzfeldt-Jakob Disease With Spinal Cord Lesions: A Case Report

**DOI:** 10.7759/cureus.103892

**Published:** 2026-02-19

**Authors:** Haruna Akanuma, Kazuaki Kanai

**Affiliations:** 1 Neurology, Fukushima Medical University School of Medicine, Fukushima, JPN

**Keywords:** autoimmune encephalomyelitis, igg index, il-6, myelopathy, sporadic creutzfeldt-jakob disease

## Abstract

Sporadic Creutzfeldt-Jakob disease (sCJD) typically presents with cognitive impairment, ataxia, visual disturbances, pyramidal and extrapyramidal signs, and myoclonus. Neuropathological involvement predominantly affects the cerebrum; however, spinal cord involvement is rare.

A 77-year-old man initially presented with numbness in the left lower limb, followed several months later by progressive lower limb paralysis and urinary dysfunction. Spinal magnetic resonance imaging (MRI) revealed spinal cord lesions, and he was admitted to a local hospital with suspected spinal cord disease. During hospitalization, his level of consciousness deteriorated, prompting transfer to our institution. Cerebrospinal fluid analysis demonstrated an elevated IgG index, making differentiation from autoimmune encephalitis challenging. However, characteristic MRI and electroencephalographic findings supported a final diagnosis of sCJD. His condition rapidly worsened, and he died approximately one month after the onset of impaired consciousness.

We report a rare case of sCJD with spinal cord involvement. The presence of spinal cord lesions may be associated with a poor prognosis and warrants clinical attention.

## Introduction

Sporadic Creutzfeldt-Jakob disease (sCJD) typically presents with cognitive impairment, ataxia, visual disturbances, pyramidal and extrapyramidal signs, and myoclonus. These manifestations often progress rapidly, with patients developing akinesia and mutism within an average of 3-4 months [1,2]. Although spinal cord involvement in sCJD is rare [1-3], its presence complicates the differential diagnosis, particularly with inflammatory myelopathies or autoimmune encephalomyelitis. This may lead to delayed recognition of prion disease or inappropriate initiation of immunotherapy, highlighting the need for careful diagnostic consideration. In both our case and previously reported cases, spinal cord lesions were identified on MRI before the appearance of characteristic prion-related findings on brain MRI. While genetic testing was not performed in the prior report, our patient was diagnosed with sCJD through genetic analysis. Reporting this case underscores the importance of considering sCJD in the differential diagnosis of unexplained spinal cord disease.

## Case presentation

**Figure 1:**
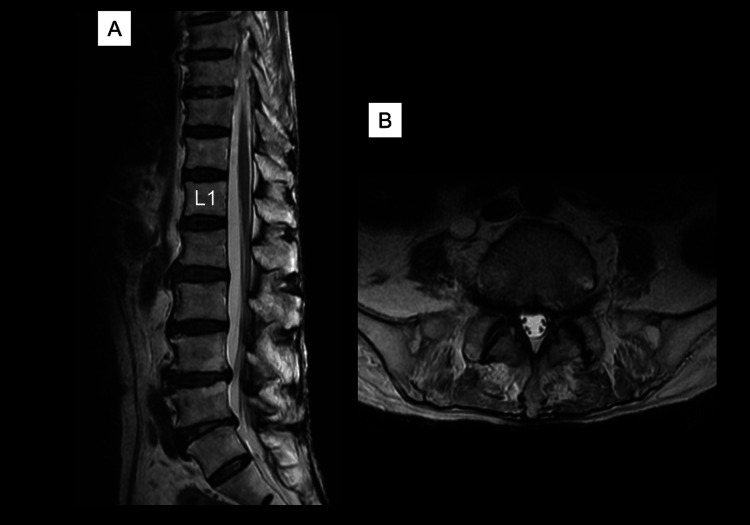
T2-weighted lumbar MRI of the patient (A) Sagittal section and (B) horizontal section (L5/S1). Spinal canal stenosis is observed.

A 77-year-old man with a history of type 2 diabetes presented with numbness in his left buttock and anterior left thigh in February 2025. An MRI of his lumbar spine taken at a nearby clinic immediately after symptoms appeared showed mild spinal canal stenosis, and he was diagnosed with spinal canal stenosis (Figure 1).

**Figure 2:**
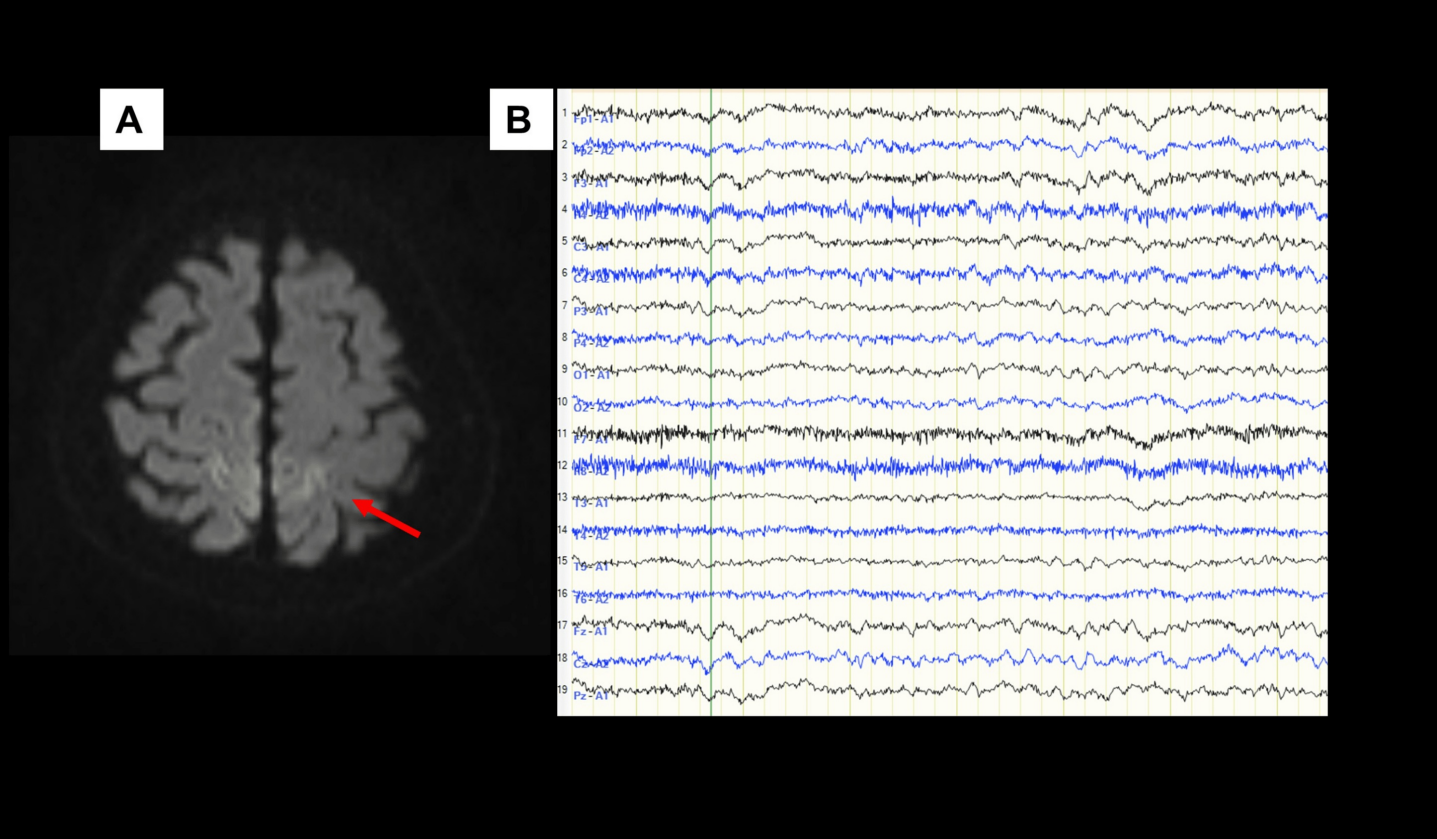
Initial MRI and EEG of the patient (A) Diffusion-weighted brain MRI performed in mid-July (axial view) suggested high signal intensity in the parietal lobe (red arrow), but it was considered to be an artifact caused by a body movement. (B) There were no notable abnormalities on the EEG in mid-August.

However, his lower limb symptoms gradually worsened, and by early July, he developed muscle weakness and urinary dysfunction. Shortly thereafter, the patient noted intermittent weakness in the left upper limb and neck discomfort, leading to hospitalization at his previous hospital in mid-July. Diffusion-weighted MRI of the brain showed increased signal intensity in both parietal cortex regions, but this was slight and could be explained by motion artifacts caused by the patient's body movement (Figure 2A).

**Figure 3:**
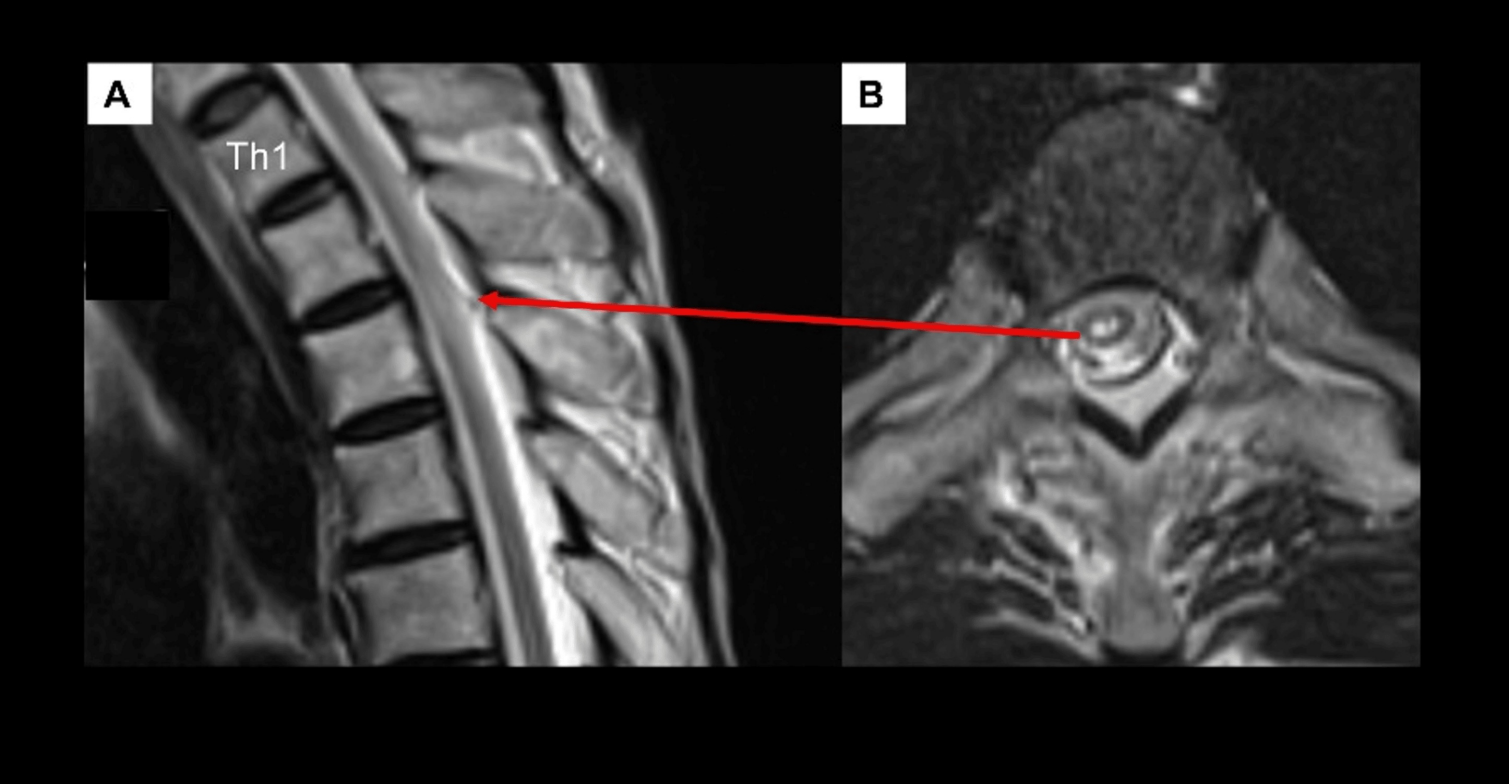
T2-weighted MRI of the thoracic spine after admission (A) Sagittal and (B) axial view of the T2-weighted MRI of the spine performed in mid-July demonstrated high signal intensity in the thoracic (Th2-Th3) region. There was no expansion of the lesion in the superior or inferior direction, and no edema or contrast effect was observed.

However, high signal intensity was also observed in the thoracic (Th2-Th3) region on T2-weighted MRI of the spine (Figure 3).

The lumbar spinal stenosis was mild, and since motor and sensory symptoms in the lower limbs were observed proximally, a thoracic spine lesion was suspected rather than a lumbar one. Cerebrospinal fluid (CSF) analysis revealed increased protein levels, but there was no increase in cell count. A diagnosis of myelopathy was made, but the muscle weakness in the lower limbs deteriorated, leading to the patient becoming bedridden, depressed, and reticent in early August. He developed impaired consciousness and was transferred to our hospital for suspected encephalomyelitis in mid-August.

At the time of transfer, the level of consciousness was E4V2M5 on the Glasgow Coma Scale. Although his eyes were open, the patient was unable to speak, but could make incomprehensible sounds. He also had myoclonus in his limbs, particularly in the right upper limb. The left upper limb muscle strength (MRC scale) was approximately 4, while the right upper limb and both lower limbs were approximately 2. There was no sensory level. Tendon reflexes in the limbs were absent, and the Babinski reflex was negative. Urinary and fecal incontinence was noted. There was no neck stiffness. Blood tests revealed hypernatremia (Na 179 mEq/L) and renal dysfunction due to dehydration. Only slow waves without notable epileptic discharges were observed on electroencephalography (EEG) (Figure 2B). Therefore, we suspected that the patient's impaired consciousness was caused by metabolic encephalopathy due to hypernatremia. We reduced sodium at a rate of approximately 5 mEq/L per day, but the patient's level of consciousness did not improve during or for several days after correction.

**Figure 4:**
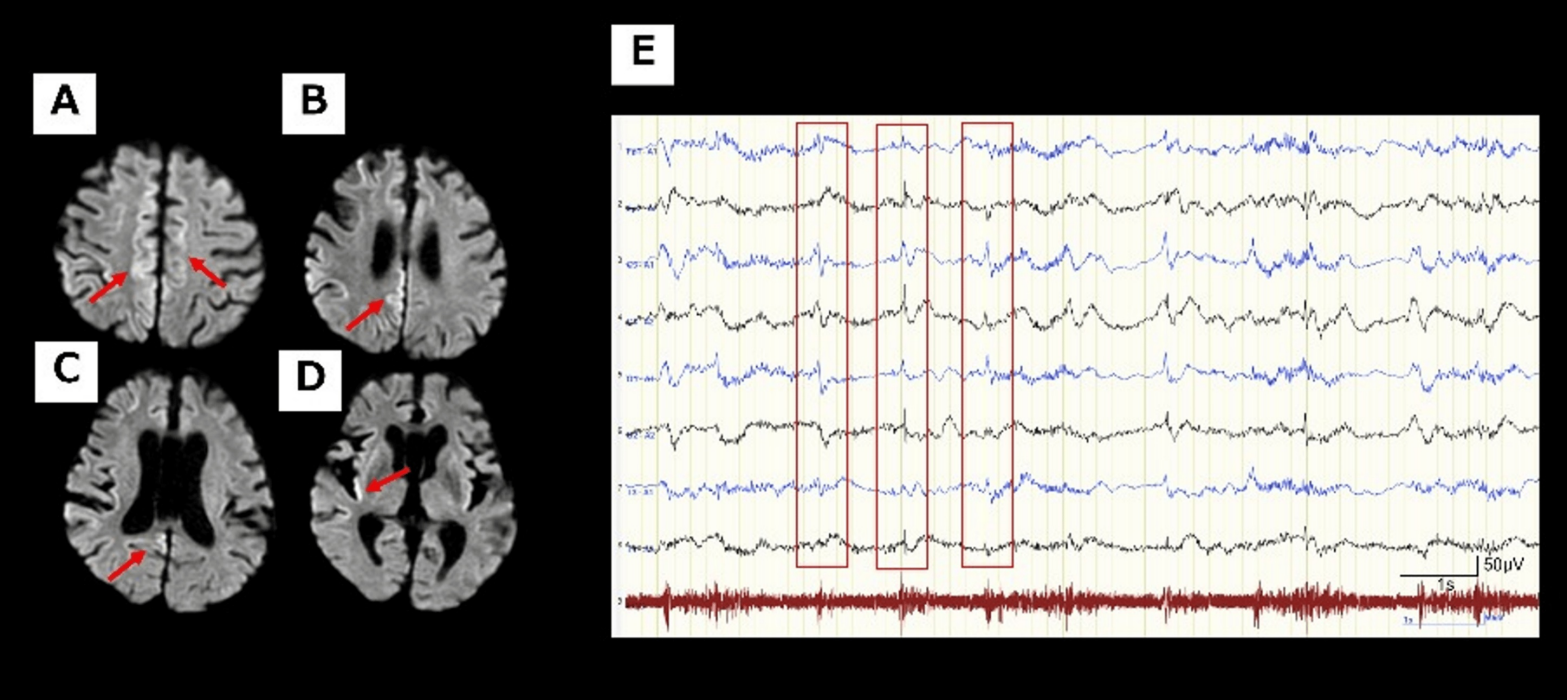
Follow-up MRI and EEG (A-D) Follow-up diffusion-weighted MRI in late August revealed clear hyperintensity in the bilateral parietal cortex (red arrow). EEG in early September revealed periodic sharp wave complexes (PSWCs) at approximately 1 Hz, typical of sCJD. (E) PSWCs were predominantly in the left hemisphere but were almost generalized (red square).

Several autoantibodies, including antinuclear antibodies, anti-DNA antibodies, anti-GAD antibodies, and anti-AQP4 antibodies, onconeuronal antibodies, were negative in the blood tests. On secondary CSF analysis, there was no increase in cell count, oligoclonal bands were absent, and polymerase chain reaction for herpes viruses was negative. Because the IgG index was high at 1.07, and IL-6 was also elevated at 25.5 pg/mL, we suspected autoimmune encephalomyelitis. However, follow-up diffusion-weighted MRI in late August revealed clear hyperintensity in the bilateral parietal cortex, with an increase in the extent of the affected area (Figures 4A-4D).

Furthermore, there were periodic sharp wave complexes (PSWCs) at approximately 1 Hz, typical of sCJD on EEG in early September (Figure 4E). By early September, the patient's responsiveness had further declined. Based on the changes observed in the MRI and EEG, we suspected a prion disease. Compressive myelopathy was ruled out based on the MRI findings. Metabolic encephalopathy due to hypernatremia was also ruled out, as there was no improvement in the patient's level of consciousness even after serum sodium levels returned to appropriate values. Paraneoplastic myelopathy was considered unlikely due to negative relevant antibodies and the absence of any apparent tumor involvement at this time. While autoimmune encephalomyelitis was difficult to rule out, it was not considered a differential diagnosis prioritized over prion disease. Therefore, CSF testing for prion disease was performed first. The CSF examination revealed markedly high levels of total tau protein (>2000 pg/mL) and 14-3-3 protein (>500 μg/mL), with real-time quaking-induced conversion (RT-QuIC) positive for prion protein (PrP). There was no family history, and no pathogenic PrP mutations were identified; polymorphism analysis showed methionine homozygosity at codon 129 and glutamic acid homozygosity at codon 219.

**Figure 5:**
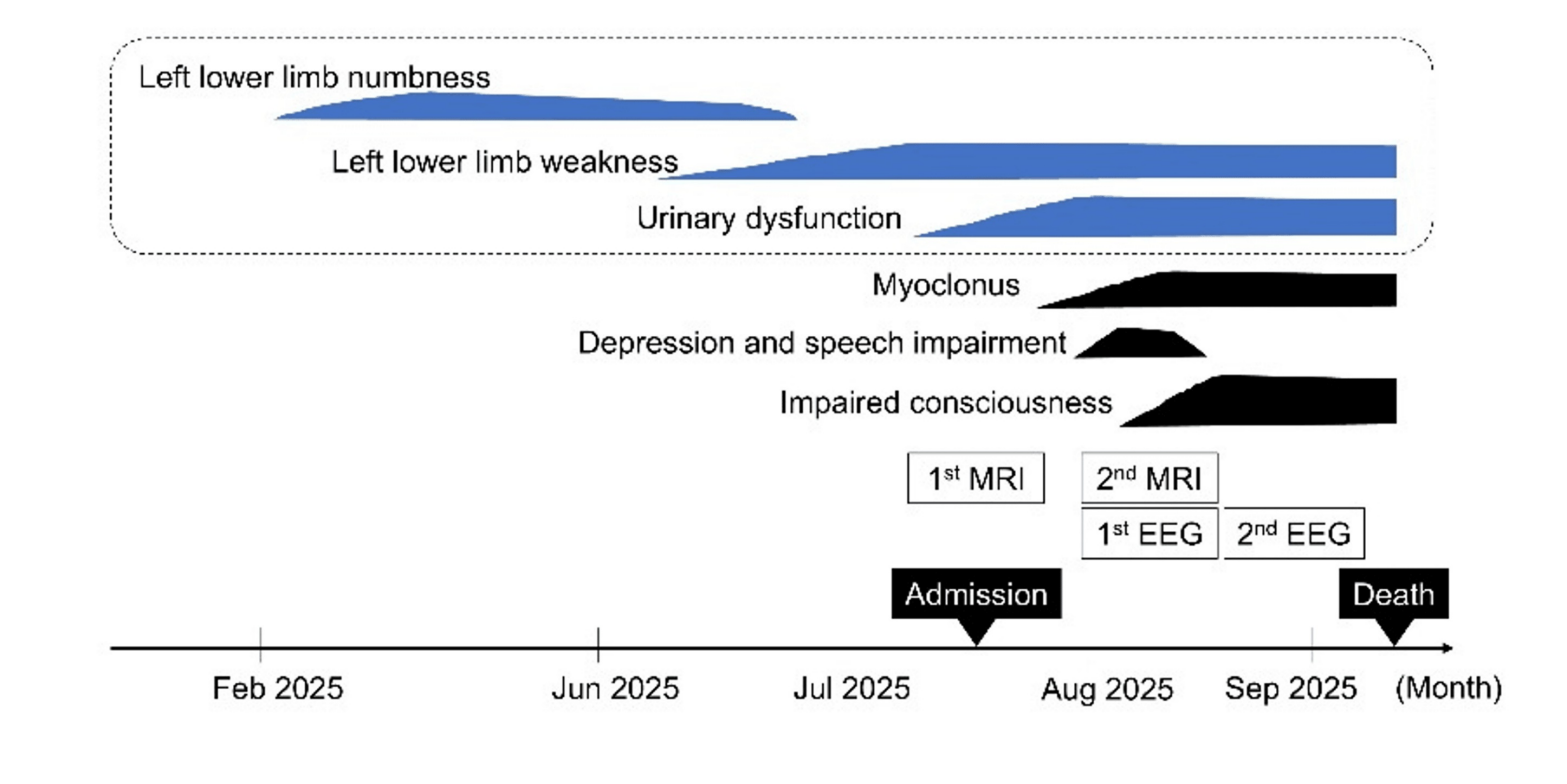
Clinical course of the patient

Based on the above, a diagnosis of sCJD was made. The patient died in mid-September due to lower gastrointestinal bleeding from a stercoral ulcer and poor respiratory status. The course of this case is summarized in Figure 5.

At the request of the patient’s family, a pathological autopsy was not performed.

## Discussion

The present case was characterized by the presence of spinal cord lesions associated with sCJD. As an autopsy was not performed, we cannot conclusively attribute the spinal cord lesions to sCJD. However, several months before the onset of impaired consciousness and myoclonus, the patient presented with symptoms suggestive of spinal cord involvement, including abnormal sensation in the lower extremities, progressive lower extremity weakness, and urinary disturbance. Therefore, we considered these symptoms to be associated with sCJD.

To date, within the scope of our search, only one case of sCJD with spinal cord lesions has been reported, and survival period was short, as in our case [3]. In experiments on animals, spinal lesions preceding brain lesions in prion diseases have been reported to be associated with poor prognosis factors [4]. Therefore, MRI abnormalities suggestive of spinal lesions in prion diseases may suggest a severe disease phenotype. In the previous report of spinal cord pathology in sCJD, diffuse abnormal PrP deposition was observed without clear spongiform degeneration [5]. The causes of prominent spinal cord symptoms and abnormal MRI findings in this case were unclear, but as PrP can spread to the spinal cord, the presence of spinal cord lesions in sCJD is reasonable.

Moreover, in sCJD, microglia are activated by PrP, leading to the production of cytokines [6]. In addition, sCJD is difficult to distinguish from autoimmune encephalitis due to an increased IgG index, CSF cell count, and protein level [7]. This case has not been pathologically proven to be non-autoimmune encephalitis. However, given the high sensitivity (around 90%) and specificity (around 98%-100%) of the RT-QUIC method [8], and combined with other test findings, the diagnosis of sCJD is considered correct.

This case report has several limitations that should be noted. The first is the absence of a pathological evaluation. This limitation applies to previously reported cases as well, including this one. In this case, the association between spinal lesions and prion disease was inferred based on the clinical course. We consider it would have been preferable to examine the involvement of spinal canal stenosis and hypernatremia pathologically as well. Second, the possibility of concomitant autoimmune encephalitis cannot be ruled out. Due to the suspicion of prion disease, specimens could not be transported to a specialized facility, preventing measurement of antineuronal surface antibodies. Only autoantibodies measurable at our facility were tested.

To address these two points, future studies should include pathological evaluation of prion disease cases suspected of having concomitant spinal cord lesions.

Through this case, we propose the following three points. First, sCJD can rarely present with spinal cord lesions preceding cognitive decline. Second, prion disease should not be excluded even in cases of myelopathy with an elevated IgG index. Third, in cases of atypical rapidly progressive myelopathy with emerging encephalopathy, prion disease should be considered, and early RT-QuIC testing should be considered.

## Conclusions

Although sCJD cases with spinal cord lesions are rare, their presence may be associated with poor prognosis, but evidence is limited to case-level data.

Even when symptoms suggestive of spinal cord diseases, such as lower limb weakness, sensory abnormalities, and urinary dysfunction, are observed, careful monitoring is warranted to determine whether the condition is progressive. If rapidly advancing impaired consciousness and cognitive decline occur, sCJD with spinal cord involvement and encephalomyelitis should be considered. Unlike the previous reports, we performed genetic testing and confirmed the diagnosis of sCJD in the present case. This demonstrates the diversity of this disease.
